# Wing Shape of Four New Bee Fossils (Hymenoptera: Anthophila) Provides Insights to Bee Evolution

**DOI:** 10.1371/journal.pone.0108865

**Published:** 2014-10-29

**Authors:** Manuel Dehon, Denis Michez, André Nel, Michael S. Engel, Thibaut De Meulemeester

**Affiliations:** 1 Laboratory of Zoology, Research Institute of Biosciences, University of Mons, Mons, Belgium; 2 Département d'entomologie, Muséum National d'Histoire Naturelle, Centre National de la Recherche Scientifique, Unité Mixte de Recherche, Paris, France; 3 Division of Invertebrate Zoology, American Museum of Natural History, New York, New York, United States of America, and Division of Entomology (Paleoentomology), Natural History Museum, and Department of Ecology and Evolutionary Biology, University of Kansas, Lawrence, Kansas, United States of America; 4 Naturalis Biodiversity Center, Leiden, the Netherlands; Ghent University, Belgium

## Abstract

Bees (Anthophila) are one of the major groups of angiosperm-pollinating insects and accordingly are widely studied in both basic and applied research, for which it is essential to have a clear understanding of their phylogeny, and evolutionary history. Direct evidence of bee evolutionary history has been hindered by a dearth of available fossils needed to determine the timing and tempo of their diversification, as well as episodes of extinction. Here we describe four new compression fossils of bees from three different deposits (Miocene of la Cerdanya, Spain; Oligocene of Céreste, France; and Eocene of the Green River Formation, U.S.A.). We assess the similarity of the forewing shape of the new fossils with extant and fossil taxa using geometric morphometrics analyses. Predictive discriminant analyses show that three fossils share similar forewing shapes with the Apidae [one of uncertain tribal placement and perhaps near Euglossini, one definitive bumble bee (Bombini), and one digger bee (Anthophorini)], while one fossil is more similar to the Andrenidae. The corbiculate fossils are described as *Euglossopteryx biesmeijeri* De Meulemeester, Michez, & Engel, gen. nov. sp. nov. (type species of *Euglossopteryx* Dehon & Engel, n. gen.) and *Bombus cerdanyensis* Dehon, De Meulemeester, & Engel, sp. nov. They provide new information on the distribution and timing of particular corbiculate groups, most notably the extension into North America of possible Eocene-Oligocene cooling-induced extinctions. *Protohabropoda pauli* De Meulemeester & Michez, gen. nov. sp. nov. (type species of *Protohabropoda* Dehon & Engel, n. gen.) reinforces previous hypotheses of anthophorine evolution in terms of ecological shifts by the Oligocene from tropical to mesic or xeric habitats. Lastly, a new fossil of the Andreninae, *Andrena antoinei* Michez & De Meulemeester, sp. nov., further documents the presence of the today widespread genus *Andrena* Fabricius in the Late Oligocene of France.

## Introduction

Bees (Apoidea; Anthophila) constitute a monophyletic group of largely pollenivorous species that rely almost exclusively on flowers for their life cycle [1]–[3]. The lineage arose from among the carnivorous apoid wasps in the late Early Cretaceous and contemporaneous with the diversification of eudicots [1], [2], [4]–[9]. Indeed, the various families of bees all appear to have originated early in the history of the clade with the constituent subfamilies and tribes diversifying at different times and in response to a variety of intrinsic and extrinsic factors [1], [9]–[11], ultimately producing a modern diversity of over 20,000 species [2]. Unfortunately, the paleontological record of bees is scant and 99% of all bee fossils are clustered in Tertiary, with most of the material spanning the Eocene and Miocene epochs [11]. Accordingly there is presently limited direct evidence of the earliest phases of bee evolution and the majority of available fossils instead provide important insights into more recent phenomena among bee evolution, such as changing patterns of biogeography and responses to Paleogene and Neogene geological events.

Four of the most important deposits with bee fossils are the Eckfeld/Messel oil shales of Germany and extensive Baltic amber deposits from the middle Eocene (∼47–44 Myr), the Florissant shale of Colorado from the Eocene-Oligocene boundary (∼34 Myr), and Dominican amber from the Early Miocene (∼19 Myr) [1], [11]–[22]. These deposits have produced sizeable bee paleofaunas compared to other deposits that produced either single specimens only (e.g. [23]–[25]) or large series of a limited number of species (e.g. [26]–[28]). Because bee fossils are relatively scarce, additional records are of significant interest to evaluate the origin of particular groups (albeit younger groups given that most higher clades are of Cretaceous age), re-evaluate hypotheses of relationship, document patterns of extinction and distribution, ascertain paleobiological associations and ancient behaviors, estimate rates of diversification/extinction, and to calibrate accurately molecular phylogenies [1], [3], [10], [11], [29].

Herein we provide the description and analysis of four newly recognized compression fossils of bees from the Upper Oligocene of Céreste (Vaucluse, France), Miocene of la Cerdanya (Spain), and Eocene shale of the Green River Formation (Utah, U.S.A.). In order to assess their taxonomic affinity with extant and extinct bee taxa, we used geometric morphometric analysis of forewing shape of 639 specimens from 50 different bee tribes and representing 188 species. Admittedly, this method is a phenetic measure of similarity and cannot determine phylogenetic relationships owing to an inability to distinguish symplesiomorphies from synapomorphies. Nonetheless, such phenetic metrics permit a more accurate determination of gross affinities for such compression fossils whereby wings are often well preserved while other anatomical traits are either incomplete, obscured, or entirely absent (e.g. [18], [27], [30]–[32]). Thus, as a first approximation geometric morphometrics provides a robust phenetic estimate of taxonomic identity and is preferable to relying on often outdated species hypotheses (e.g. [21]). In fact, it has been emphasized that systematic melittology is in need of exploring new methods and character systems for both the circumscription of taxa and the recovery of genealogical relationships (e.g. [33], [34]), and the methods employed herein are one step in that direction.

## Materials and Methods

### Description, terminology and repositories

The morphological terminology of the bee wings and bodies follows that of Engel (2001) [1], while the general classification follows that of Michener (2007) [2]. The two fossils from Cereste and the single specimen from Cerdanya are deposited in the Museum National d'Histoire Naturelle (Paris, France), while the fossil from Green River Formation is deposited in University of Kansas Natural History Museum (Lawrence, Kansas, USA). Representative bee specimens were sampled from the following collections: Laboratoire de Zoologie (University of Mons, Belgium); Département d'Entomologie Fonctionnelle et Evolutive (University of Liège, Belgium); Musée Royal de l'Afrique Centrale (Tervuren, Belgium); Musée de l'Institut Royal des Sciences Naturelles (Brussels, Belgium); Natural History Museum (London, United Kingdom); and Naturalis Biodiversity Center (Leiden, Netherlands).

No permit were required for the described study, which complied with all relevant regulations.

### Nomenclatural Acts

The electronic edition of this article conforms to the requirements of the amended International Code of Zoological Nomenclature, and hence the new names contained herein are available under that Code from the electronic edition of this article. This published work and the nomenclatural acts it contains have been registered in ZooBank, the online registration system for the ICZN. The ZooBank LSIDs (Life Science Identifiers) can be resolved and the associated information viewed through any standard web browser by appending the LSID to the prefix “http://zoobank.org/”. The LSID for this publication is urn:lsid:zoobank.org:pub:E4FE4837-1579-4BC7-BD43-9E08087B0351. The electronic edition of this work was published in a journal with an ISSN, and has been archived and is available from the following digital repositories: PubMed Central, LOCKSS.

### Geological settings

The Green River Formation is exposed in a variety of sedimentary basins in northeastern Utah, southern Wyoming, and northwestern Colorado. One such basin is that of the Uinta, which spans between Utah and Colorado and represents a shallow paleolake nestled among mountains that persisted for ∼20 Myr (latest Paleocene to Late Eocene). The Parachute Creek Member in this basin mainly formed during the middle Eocene (∼47 Myr) [35], [36], and is where one of the specimens analyzed herein originated. The outcrops contain many insect compressions in oil shale. The flora found in this section of the basin suggests a tropical to subtropical climate with a distinct dry season [37], and occurred at a paleoelevation of ∼1500–2900 m [38].

The Oligocene lacustrine beds (‘calcaire de Campagne-Calavon’) of Céreste are exposed along the northern margin of the Lubéron mountain and represent a shallow paleolake. These beds were long considered as Rupelian (∼30 Myr) [39], but the most recent study of Gregor (2002) [40] rather suggests a Late Oligocene age, the exact age of this formation remains controversial. The vertebrates fossils discovered would suggest a rather semi-arid palaeoenvironment [41], while the rich flora implies a mixed-mesophytic forest [40]. The high diversity and abundance of the entomofauna with thousands of different species belonging to most orders also supports a forested palaeoenvironment surrounding a lake. Moreover, observations from the Bibionidae (Diptera) imply a warmer episode than the latest Oligocene of Aix-en-Provence [42]. The rather reduced aquatic fauna reflects the presence of waters of poor quality rather than a dry climate (A.N. pers. obs.). Apoidea are not frequent in these beds most specimens belong to *Apis* [26].

The Late Miocene lacustrine beds (Vallesian – Turonian, ∼10 Myr) of the Spanish Cerdanya are located around the small town of Bellver. They correspond to an association of fine-grained terrigenous sequences and lacustrine diatomites with levels of diagenetic phosphates [43]. They are supposed to correspond to a deep water palaeolake. The palaeoclimate of this mountain lake (∼1100 m) was warmer than today [44]. The flora and entomofauna are very abundant and diverse, with lists of the fossil insects currently described given by Peñalver-Molla *et al.* (1999) [45] and Arillo (2001) [46]. Apoidea are rather frequent in these beds, although nearly all specimens belong to the genus *Apis* [26].

### Dataset and morphometric analyses

We performed geometric morphometric analyses to assess the taxonomic affinity of the four new fossils. Geometric morphometrics is a core of procedures providing quantification of the global shape of a structure [47]–[49]. Geometric morphometrics can provide tools in paleontology for diagnosing fossil taxa at different levels, and for estimating their taxonomic affinities with extant taxa [31]–[32]. Phylogenetic relationships cannot be determined owing to an inability to distinguish symplesiomorphy from synapomorphy, but remains one of the most robust methods for ascertaining gross affinities of fragmentary or problematic fossils (e.g. [18], [31], [32]). Thus, while taxa can be robustly assigned to higher group any supraspecific taxa established based on geometric morphometrics run the risk of creating paraphyletic groups and therefore should still be tested in future studies by more complete material and cladistic analyses of additional character systems (e.g., non-wing traits).

Taxonomic affinities of the four new fossils discussed herein were based on their forewing shape. Wings have many methodological advantages: 2D structure, rigidity, species specificity, and typically excellent preservation in fossil specimens [50]. Moreover, wing veins and their intersections are unambiguously homologous among bees in taxa with three submarginal cells in their forewings [2], [51]. Most importantly, for all of the specimens analyzed herein the wings were preserved flat (i.e., not folded or crumpled) and without postmortem tectonic distortion, thereby permitting meaningful comparison with extant species in the absence of retro-deformation (i.e., retrofitting a distorted wing as preserved back to its presumed living orientation of veins).

Given that all of the new fossil specimens exhibit three submarginal cells, we sampled broadly across tribes of bees and apoid wasps with the same number of submarginal cells, and with a maximum of 20 specimens per tribe. We attempted to maximize the morphological diversity of our dataset by selecting four species per tribe and five specimens per species, when possible. In addition, we included 14 extinct species with well-preserved forewings and robust taxonomic assignments, ideally based on a broad suite of available characters [Apidae: Apinae: *Paleohabropoda oudardi* Michez & Rasmont, 2009 (Anthophorini); *Bombus randeckensis* Wappler & Engel, 2012 (Bombini); *Electrapis meliponoides* (Buttel-Reepen, 1906), *E. krishnorum* (Engel, 2001), *Protobombus basilaris* Engel, 2001, *P. hirsutus* (Cockerell, 1908) and *Thaumastobombus andreniformis* Engel, 2001 (Electrapini); *Melikertes stilbonotus* (Engel, 1998), *Melissites trigona* Engel, 2001 and *Succinapis goeleti* Engel, 2001 (Melikertini); *Anthophorula persephone* Engel, 2012 (Exomalopsini); *Eufriesea melissiflora* (Poinar, 1998) (Euglossini); Halictidae: Halictinae: *Halictus petrefactus* Engel & Peñalver, 2006 (Halictini); *Electrolictus antiquus* Engel, 2001 (Thrinchostomatini)]. We assembled a global reference dataset of 632 female specimens representing 10 families, 21 sub-families, 39 tribes, 117 genera, and 188 species of Anthophila and apoid wasps (Table 1). As two of the four new fossils were attributed to the tribes Bombini and Anthophorini, we also assessed their generic attribution based on the dataset described in Wappler *et al.* (2012) [18] (n = 336 specimens) and Michez *et al.* (2009) [31] (n = 37 specimens) respectively (Tables 2 and 3 respectively).

**Table 1:** Reference data set for the geometric morphometric analysis.

Family	Subfamily	Tribe	N1	N2
**Anthophila**				
Andrenidae	Andreninae		4	20
	Oxaeinae		5	5
	Panurginae	Melitturgini	3	7
		Protandrenini	2	4
Apidae	Apinae	Ancylaini	4	16
		Anthophorini	8	20+1†
		Apini	4	20
		Bombini	5	20+1†
		Centridini	5	20
		Electrapini†	5	6
		Emphorini	8	20
		Ericrocidini	6	20
		Eucerini	4	20
		Euglossini	5	20+1†
		Exomalopsini	3	5+1†
		Isepeolini	1	1
		Melectini	6	20
		Melikertini†	3	3
		Osirini	3	7
		Protepeolini	2	2
		Tapinotaspidini	5	9
		Tetrapediini	3	7
	Nomadinae	Brachynomadini	2	2
		Epeolini	5	9
		Nomadini	4	20
	Xylocopinae	Ceratinini	5	20
		Xylocopini	4	20
Colletidae	Colletinae	Colletini	5	20
		Paracolletini	1	5
	Diphaglossinae	Caupolicanini	7	20
		Diphaglossini	4	2
		Dissoglottini	1	1
Halictidae	Halictinae	Augochlorini	4	20
		Caenohalictini	5	20
		Halictini	6	20+1†
		Sphecodini	5	20
		Thrinchostomatini	5	20+1†
	Nomiinae		7	20
	Nomioidinae		3	11
	Rophitinae		5	20
Megachilidae	Fideliinae	Fideliini	4	20
Melittidae	Meganomiinae		2	10
	Melittinae	Melittini	2	20
**Stenotritidae**			2	2
Sphecid Wasp				
Ampulicidae	Ampulicinae	Ampulicini	2	10
Crabronidae	Larrinae		1	5
	Philanthinae		1	5
Sphecidae	Ammophilinae		1	5
	Sphecinae		1	5

This sampling includes 632 specimens from 188 species, 117 genera, 39 tribes, 21 subfamilies, and 10 families of Apoidea. N1 = number of species. N2 = number of specimens.

† = extinct species.

**Table 2:** Groups sampled for geometric morphometric analyses in Wappler *et al.* (2012).

Taxon	Number of species	Number of specimens
**Bombini**		
Genus *Bombus*		
Subgenus *Alpigenobombus*	4	13
Subgenus *Alpinobombus*	4	14
Subgenus *Bombias*	2	10
Subgenus *Bombus*	10	19
+*B.* (*Bombus*) *randeckensis* †	1	1
Subgenus *Cullumanobombus*	11	16
Subgenus *Kallobombus*	1	9
Subgenus *Megabombus*	16	36
Subgenus *Melanobombus*	7	13
Subgenus *Mendacibombus*	8	14
Subgenus *Orientalibombus*	2	6
Subgenus *Pyrobombus*	11	24
Subgenus *Sibiricobombus*	7	17
Subgenus *Subterraneobombus*	10	32
Subgenus *Thoracobombus*	29	93
**Centridini**		
Genus *Centris*	1	3
**Anthophorini**		
Genus *Anthophora*		
Subgenus *Anthophora*	1	3
Genus *Habropoda*	1	3
Genus *Pachymelus*		
Subgenus *Pachymelus*	1	3
Genus *Paleohabropoda* †	1	1

This sampling includes 328 bee specimens from three apine tribes [Bombini (n = 316), Centridini (n = 3), and Anthophorini (n = 9)].

**Table 3:** Groups sampled for geometric morphometric analyses in Michez *et al.* (2009).

Taxon	Number of sampled specimens
**Anthophorini**	
*Amegilla albigena* Lepeletier 1841	3
*Am. quadrifasciata* de Villers 1789	3
*Anthophora aestivalis* (Panzer 1801)	3
*A. bimaculata* (Panzer 1798)	3
*A. plumipes* (Pallas 1772)	3
*A. quadrimaculata* (Panzer 1798)	3
*Deltoptila elefas* (Friese 1917)	3
*Elaphropoda moelleri* Lieftinck 1966	1
*E. percarinata* (Cockerell 1930)	1
*Habropoda tarsata* (Spinola 1838)	3
*H. zonatula* Smith 1854	2
*Habrophorula nubilipennis* (Cockerell 1930)	1
*Pachymelus ocularis* Saussure 1890	3
*P. radovae* Saussure 1890	1
*P. unicolor* Saussure 1890	3
*Paleohabropoda oudardi* Michez & Rasmont 2009	1

This sampling includes 37 specimens representing 16 species and 8 genera of Apinae (Apidae).

**Figure 1:**
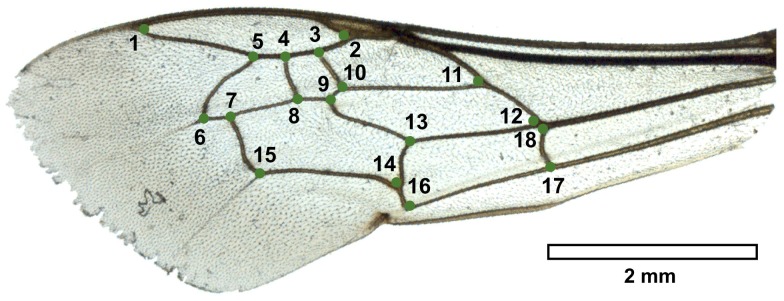
Left forewing of *Melitta leporina* with the 18 landmarks selected to describe the shape.

Forewings were photographed using an Olympus SZH10 microscope coupled with a Nikon D200 camera. Photographs were input to tps-UTILS 1.56 [52]. The left forewing shape of the 632 specimens and of the four new fossils were captured from photographs by digitizing two-dimensional Cartesian coordinates of 18 landmarks placed on the wing veins (Fig. 1) with tps-DIG v2.17 [53]. The 636 landmark configurations of the reference dataset were scaled, translated and rotated against the consensus configuration using the GLS Procrustes superimposition method to remove all of the non-shape differences and to separate the size and shape components of the form [47], [54]. The superimposition was performed using R functions of the package “geomorph” [55]. The aligned landmark configurations were projected into the Euclidean space tangent to the curved Kendall's shape space to aid further statistical analyses. The closeness of the tangent space to the curved shape space was tested by calculating the least-squares regression slope and the correlation coefficient between the Procrustes distances in the shape space with the Euclidean distances in the tangent space [56]. This variation amplitude of our dataset was calculated with tps-SMALL v1.25 [57].

As some cross-veins were difficult to define on the body fossil of *Euglossopteryx*, the landmarks positions were checked by three authors (MD, DM and TD) and by performing a superimposition of the right forewing on the left forewing to accentuate the veins. Landmarks coordinates resulting from digitalization of wing shape of Anthophorini [31], Bombini [18], four new fossils and 632 original specimens are available in Tables S1, S2, S3 and S4 respectively. The entomological collections from where the specimens were loaned are available in Table S4.

### Validation of shape discrimination at different taxonomic levels

Prior to assignment of the four new fossils, shape variation within the reference dataset and discrimination of the different taxa was assessed by Linear Discriminant Analyses (LDA) of the projected aligned configuration of landmarks. These analyses were performed at suprafamily (i.e. bees *versus* apoid wasps), family, subfamily, and tribal levels as *a priori* grouping by using the software R version 3.0.2 [58]. The effectiveness of the LDA for discriminating taxon was assessed by the percentages of individuals correctly classified to their original taxon (hit-ratio, HR) in a leave-one-out cross-validation procedure based on the posterior probabilities of assignment. Given the observed scores of an “unknown”, the posterior probability (pp) equals the probability of the unit to belong to one group compared to all others. The unit is consequently assigned to the group for which the posterior probability is the highest [59].

### Assignment of the bee fossils

Taxonomic affinities of the new fossils were first assessed based on their score in the predictive discriminant space of shapes. After superimposition of the 636 landmark configurations (i.e. corresponding to the reference dataset and the four new fossils), aligned coordinates of the 632 specimens from the reference dataset were used to calculate the LDA. A unique superimposition of both the reference dataset and the assigned specimens is sometimes disregarded while it is of primary importance because GLS Procrustes superimposition is sampling dependent. We included a posteriori the four new fossil specimens in the computed LDA space as “unknown” specimens and calculated their score. Assignments of the new fossils were estimated by calculating the Mahalanobis Distance between “unknowns” and group mean of each taxa (Table S5). We also calculated posterior probabilities of assignment to confirm the assignment to one taxon (Table S5). Assignments of the new fossils were performed in four consecutive analyses corresponding to different taxonomic levels of *a priori* grouping: suprafamily (i.e. apoid wasps *versus* bees), family, subfamily, and tribe.

In order to further assess the generic taxonomic affinity of the two new fossils associated with the tribes Bombini and Anthophorini, the reference datasets assembled in Wappler *et al.* (2012) [18] and Michez *et al.* (2009) [31] were used. PCA were computed to visualize shape affinities between the fossils and the genera in question.

## Results

### Morphometric analysis

The regression coefficient between the Procrustes distances and the Euclidean distances is close to 1 (0.9999). This means that the linear tangent space closely approximates the shape space, thereby permitting us to be confident in the variation amplitude of our dataset.

#### Shape variation within the reference dataset

In the morphometrics space defined by the LDA based on suprafamily *a priori* grouping, the two groups are perfectly discriminated: all 602 bee specimens and the 30 apoid wasp specimens are assigned to their original group by the cross-validation procedure. In the LDA space with family *a priori* grouping, the 10 families are well isolated from each other and only 10 specimens are not assigned to their original group by the cross-validation procedure, accounting for a global HR of 98.4%. The Ampulicidae, Crabronidae, Halictidae, Sphecidae, and Stenotritidae show a HR of 100%. The Andrenidae, Apidae, Colletidae, Megachilidae, and Melittidae have from a single to three specimens misclassified compared to the original classification, accounting for a minimal HR of 91.7% (Andrenidae). Among the 10 misclassified specimens, nine specimens show poorly supported assignment (i.e., with low posterior probability) and should be considered as dubiously classified. Moreover, the misclassified specimens belong to different species and groups, rejecting the hypothesis of poorly discriminated taxa that could affect the assignment. All of the 14 described fossils are attributed to their original family. As observed at the familial level, subfamily discrimination by LDA was effective, with a cross-validated HR of 97.5% (i.e., 16 misclassified specimens), and 14 of the 21 subfamilies account for a HR of 100% (Table S6). Five subfamilies have a HR between 95% and 99%, and two subfamilies have a HR lower than 95% (i.e., Diphaglossinae and Panurginae, Table S6). The low HR observed in Diphaglossinae and Panurginae are due to the large shape difference observed among tribes of these subfamilies where the intertribal shape differences are as large as subfamilial differences (e.g., among the Melitturgini and Protandrenini). It would be interesting in the future to include further tribes, genera, and species in these subfamilies so as to see if a denser sampling of taxa might alleviate some of these deficiencies. Among the 16 misclassified specimens, nine specimens show poorly supported assignment (i.e., with low posterior probability) and should be considered as dubiously classified. All of the 14 described fossils are attributed to their original subfamily. The 49 groups defined at the tribal level are well discriminated in the LDA, with 32 misclassified specimens, accounting for a global HR of 94.9%. Among the 49 groups, cross-validation cannot be performed on two groups because these each include a single specimen, 31 groups account for a HR of 100%, seven groups show HR between 90% and 99%, and nine groups have a HR lower than 90% (Table S7). Due to sampling size within groups, the HR drastically drops when one or two specimens are misclassified. This is the case for 14 groups among the 16 with HR lower than 100%. Two groups are poorly discriminated in the LDA: Ancylaini and Emphorini. Several described fossils are not attributed to their original tribe, particularly the Electrapini and Melikertini, but this is perhaps owing to the generally plesiomorphic nature of their wing venations. Moreover, some bee tribes (8 tribes with 3 submarginal cells out of 47 reported in [2]) are not included in the analyses and so it is perhaps not surprising that the analyses would have some difficulties at the tribal level for some groups. Nonetheless, the overall results demonstrate a remarkable fidelity in accurately placing particular taxa. Thus, the cross-validation assignments (Tables S6, S7) allow us to be confident in the group discrimination at most higher taxonomical levels. The specimens' scores along the LDs (for the following taxonomic levels: suprafamily, family, subfamily and tribe) are available in the Table S8.

#### A posteriori assignment of the fossils

Taxonomic affinities are detailed for each four new fossils. The Green River specimen is assigned to Anthophila (MD = 1.09; pp = 1), to Apidae (MD = 7.43; pp = 0.999), to Apinae (MD = 13.01; pp = 0.999), and to Euglossini (MD = 20.02; pp = 0.999) in the respective LDA. The Cerdanya fossil is assigned to Anthophila (MD = 0.09; pp = 1), to Apidae (MD = 1.70; pp = 0.999), to Apinae (MD = 3.98; pp = 0.999), and to Bombini (MD = 4.82; pp = 1) in the respective LDA. The first fossil from Céreste is assigned to Anthophila (MD = 1.40; pp = 1), to Apidae (MD = 6.97; pp = 0.999), to Apinae (MD = 9.19; pp = 0.999), and to Anthophorini (MD = 12.73; pp = 0.998) in the respective LDA. Lastly, the second fossil from Céreste is assigned to Anthophila (MD = 1.25; pp = 1), to Andrenidae (MD = 5.12; pp = 0.926), and to Andreninae (MD = 6.74; pp = 0.999) in the respective LDA. Given that no tribes were defined within the subfamily Andreninae, the “Andreninae” group was used for the tribal a priori grouping. The andrenine fossil was assigned to this group in the fourth LDA (MD = 9.30; pp = 1).

**Figure 2:**
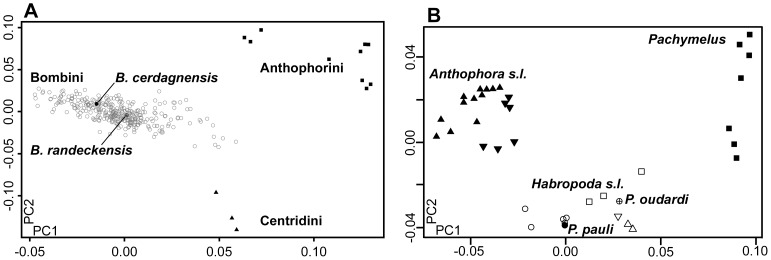
Generic taxonomic affinity of the two new fossils associated with the tribes Bombini and Anthophorini. A. Ordination of the Bombini and *Bombus cerdanyensis* sp. nov. along the first two axes of the PCA (PC1 = 32% and PC 2 = 19%). B. Ordination of the Anthophorini and *Protohabropoda pauli* gen. nov. sp. nov. along the first two axes of the PCA (PC1: 59%; PC2: 16%).

Taxonomic affinities of the bombine fossil from the lacustrine beds of Cerdanya were also assessed based on the reference dataset described in Wappler *et al.* (2012) [18] (Table 3). In the morphometric space defined by the PCA, this fossil is undoubtedly clustered with the genus *Bombus* (Fig. 2a). A-posteriori assignment of the fossil in the discriminant shape space based on the subgenera of *Bombus* does not allow a reliable subgeneric attribution.

Taxonomic affinities of the anthophorine fossil from Céreste were also assessed based on the reference dataset described in Michez *et al.* (2009) [31] (Table 3). In the morphometric space defined by the PCA, this fossil is undoubtedly clustered with the group of the *Habropoda sensu lato*, and more particularly with the genus *Habropoda* (Fig. 2b).

As mentioned previously, all of the specimens have three submarginal cells and therefore likely do not belong to the Xeromelissinae, Hylaeinae, or Euryglossinae (Colletidae), Dasypodainae (Melittidae), Lithurginae and Megachilinae (Megachilidae), and various tribes of Apidae (e.g., Allodapini, Ammobatini, Ammobatoidini, Biastini, Boreallodapini, Caenoprosopidini, Ctenoplectrini, Neolarrini, and Townsendiellini) which have 2 submarginal cells.

The positions of all 4 fossils in the shape space (PCA) and in the discriminant space (LDA) are available at http://beefossil.naturalis.nl/.

### Systematic paleontology

Family: Apidae Latreille 1802

Subfamily: Apinae Latreille 1802

Clade: Corbiculata Engel, 1998

Tribe: incertae sedis

***Genus*** **Euglossopteryx** ***Dehon & Engel gen. nov.***

urn:lsid:zoobank.org:act:BC00394F-1FA5-4787-86E8-3D40E210BBB0

Type species. *Euglossopteryx biesmeijeri* De Meulemeester, Michez, & Engel, new species.

Diagnosis. Female of robust body form (body habitus is that of small *Bombus*, *Melipona*, *Protobombus*, or *Euglossella*), apparently without metallic coloration (although coloration is poorly preserved and may reflect only relative shades of areas as they were in life); metatibia corbiculate, with long fringe of setae along border, not dramatically expanded posteriorly (in this respect differing from non-parasitic Euglossini); forewing with first submarginal cell longest, shorter than combined lengths of second and third submarginal cells; pterostigma apparently relatively small (incompletely preserved but distinctly not the more triangular or elongate forms of some Meliponini; more similar to form observed in Bombini and Euglossini); marginal cell long, closed apically, apex acutely rounded (not open as in Meliponini nor greatly elongate as in Apini); 1m-cu meeting second submarginal cell, not strongly angulate (more distinctly angulate in many Euglossini and Bombini, more similar to some Electrapini or Melikertini in this respect); membrane not infuscate nor papillate (differing in this respect from most Bombini); first abscissa of Rs relatively straight (similar in this respect to many Euglossini, some Electrapini, and some Melikertini).

Etymology. The new genus-group name is a combination of *Euglossa*, type genus of the Euglossini and the tribe to which the fossil showed its greatest affinity, and *pteryx* (Greek, meaning, “wing”), and references to the *Euglossa*-like venation of the fossil. The name is feminine.

**Euglossopteryx biesmeijeri** ***De Meulemeester, Michez & Engel sp. nov.***

urn:lsid:zoobank.org:act:2E0EF5AB-0B1B-4461-9A5F-7E3828D84B30

Holotype. Female; Division of Entomology (Paleoentomology), University of Kansas Natural History Museum, Lawrence, Kansas, U.S.A.

Type strata and locality. Eocene, oil shale deposit, Parachute Creek Member of the Green River Formation, Uinta Basin, Utah, U.S.A.

Diagnosis. As for the genus (*vide supra*).

**Figure 3:**
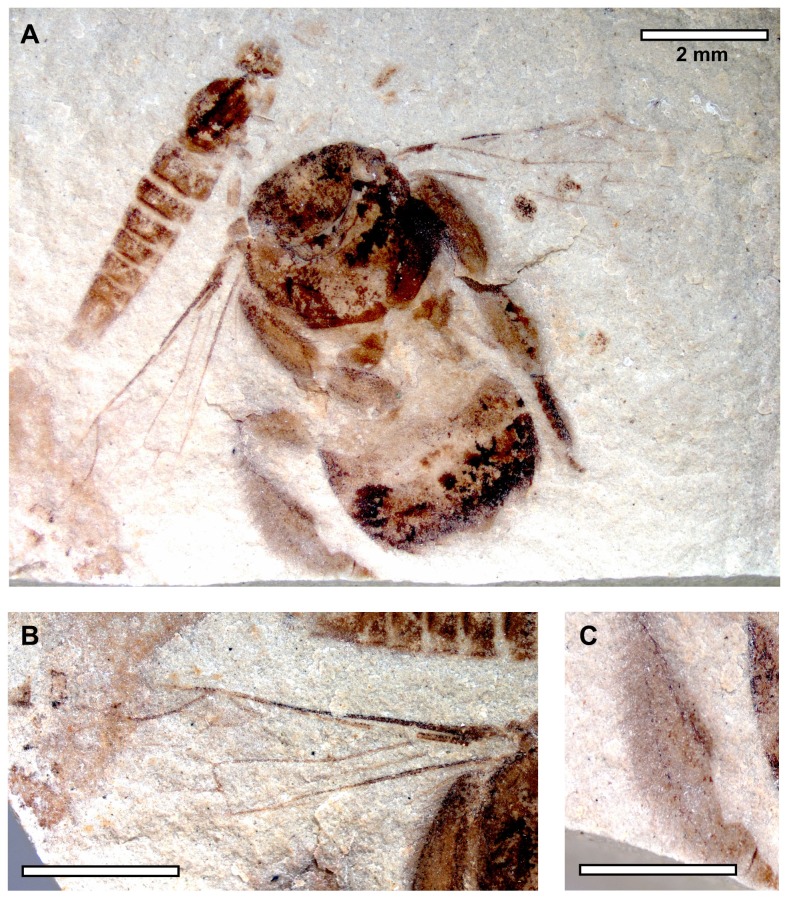
*Euglossopteryx biesmeijeri* gen. nov sp. nov. Dorsal view (photographs by N. J. Vereecken).

Description. *Female* (Fig. 3). A female bee missing the head preserved with forewings outstretched (albeit slightly obliquely) with dorsoposterior oblique view of mesosoma, dorsal view of metasoma (slightly detached from mesosoma), and the hind and midlegs preserved alongside the body (left hind leg more outstretched and with posterior surface of metatibial corbicula evident. Wings preserved flat, with small portions of hind wings visible, leading edge of forewings not preserved or partially folded (thus pterostigma is foreshortened). Mesosomal length 4.00 mm as preserved (orientation is not a direct dorsal view), intertegular distance 2.00 mm. Tegulae preserved in oblique dorsal view, apparently ovoid with slightly angulate posterior inner border (much as in various corbiculate groups, including Euglossini: e.g., see tegular shape as depicted in Hinojosa-Díaz *et al.* (2011) [60]; posterior half of mesoscutum visible, 1.66 mm long as preserved; mesoscutellum 0.09 mm long; metanotum 0.32 mm long; propodeum 1.56 mm long as preserved across dorsal and posterior surfaces, dorsal surface apparently quite short, apparently no overhung by mesoscutellum or metanotum, propodeal pit present, narrow; pilosity of mesosoma generally scattered, short, only mesoscutum and mesoscutellum with setae over entire surfaces. Left mesofemur 2.49 mm long; mesotibia 1.75 mm long as preserved (incomplete); right mesofemur 2.57 mm long; mesotibia 2.14 mm long; mesotarsus 2.53 mm long; pretarsal claw 0.11 mm long (the state of preservation does not allow us to know if it is toothed or simple); left metafemur 1.30 mm long as preserved (incomplete); metatibia 4.23 mm long as preserved, slightly oblique, metatibia distinctly corbiculate, fringed by long setae; right metacoxa (incomplete) 0.31 mm long as preserved; metatrochanter 0.39 mm long as preserved (incomplete); left metacoxa 0.32 mm long as preserved (incomplete); metatrochanter 0.46 mm long as preserved (incomplete); right mesobasitarsus 1.91 mm long, 0.26 mm wide; right mesomediotarsus 0.68 mm long, 0.13 mm wide; right mesobasitarsus shorter than right mesotibia; black punctures mark base of setae on metatrochanters, mesofemora, mesotibiae, metafemora, and metatibiae. Left and right forewing each 8 mm long; three submarginal cells (i.e., 1rs-m present); first submarginal cell 1.23 mm long (as measured from origin of Rs+M to juncture of r-rs and Rs), 0.47 mm high (as measured from Rs+M to pterostigma); second submarginal cell 0.99 mm long (as measured from juncture of Rs+M and M to juncture of Rs and 1rs-m), 0.43 mm high (as measured from midpoint on M between 1m-cu and 1rs-m to juncture of r-rs and Rs); third submarginal cell 0.84 mm long (as measured from juncture of 1rs-m and M to juncture of M and 2rs-m), 0.41 mm high (as measured from juncture of M and 2m-cu to juncture of 2rs-m and Rs); first medial cell 2.47 mm long (measured from juncture of M+Cu and Cu to juncture of 1m-cu and M), 0.68 mm high (measured from juncture of M and Rs+M to midpoint on Cu between M+Cu and 1m-cu); pterostigma small, 0.2 mm long; marginal cell 2.04 mm long, apex acutely pointed; 2m-cu not entirely visible; 1m-cu joining second submarginal cell near its apex, not strongly angulate, relatively straight in basal half, then arched to meet M; first abscissa of Rs straight, 1.39 mm long. Metasomal length 5.43 mm as preserved (apical most segments slightly recessed into preceding segments; likely a postmortem factor), width 4.67 mm as preserved, with only scattered setae present apically.

#### Male

Unknown.

Etymology. The specific epithet is a patronym honoring Prof. Jacobus Biesmeijer, scientific director at Naturalis Biodiversity Centre and professor of Functional Biodiversity at the University of Amsterdam, a leading authority on pollinator-plant interactions and pollinator declines, and for his inspiration of many melittologists throughout the world. Prof. Biesmeijer is acknowledged for his career as a leading scientist who stimulates bee research.

Comments. As noted in the Results, *Euglossopteryx biesmeijeri* gen. nov. sp. nov. was confidently assigned to the Apinae. Moreover, aside from forewing shape, the specimen shows other traits of bees and apines, in particular, such as branched setae, an expanded tarsus, and even the posterior surface of a corbiculate metatibia (Fig. 3). Given that *E. biesmeijeri* exhibits pollen-collecting structures, it is clear that the species is not cleptoparasitic, thereby excluding the various cuckoo bee tribes and genera [2].

The most revealing structure as to the affinities of *E. biesmeijeri* is the presence of a metatibial corbicula, a unique synapomorphy of the corbiculate tribes in the Apinae. The clade Corbiculata comprises of four extant tribes (Apini, Bombini, Euglossini, and Meliponini) and three extinct groups (Melikertini, Electrapini, and Electrobombini) [1], [14]. *E. biesmeijeri* can be excluded from the Meliponini and Apini as it does not have the dramatically reduced wing venation of the former, nor the uniquely modified and extended marginal cell (i.e., nearly four times as long as the distance from its apex to the wing tip) of the latter. The forewings of *E. biesmeijeri* do not possess alar papillae, suggesting that the species was not a bombine. The pterostigma is no longer that the prestigma, unlike the fossil tribes Melikertini, Electrapini, and Electrobombini, although admittedly there remains much to be learned about wing form diversity in these lineages. The 1m-cu vein is strongly angulate unlike Electrapini and Electrobombini and the body size was likely greater than the known melikertines, although body size is not necessarily a diagnostic feature of this group. The metatibia is not as grossly expanded as is true among the Euglossini. Overall the shape of the forewing is most similar to Euglossini (refer to Results, above), but this may very likely be a shared plesiomorphy given that the wing venation of Euglossini is very generalized relative to other corbiculate tribes and euglossines are frequently recovered as the basal lineage of the clade [14], [61]. More importantly, the corbiculate clade is quite ancient, extending at least into the Late Cretaceous [62] and with significant levels of extinction [1], [14], leaving only four, morphologically-isolated extant tribes. It is exactly for such a scenario that early fossils will be the most critical for resolving controversies over relationship, in this case meaning those taxa from the latter part of the Cretaceous, and there will likely be a larger number of Paleogene taxa that are difficult to assign as to tribe.

An affinity between *E. biesmeijeri* with euglossines is certainly plausible given the distribution and ecology of the latter. Moreover, orchid bees were certainly present in the Tertiary of North America as evidenced by fossils in Early Miocene amber of the region (e.g. [63], [64]). We are confident that *E. biesmeijeri* is near to the Euglossini and may even belong to the crown group, but this will require further and more completely preserved material and it could be a stem-group euglossine. Future studies encompassing a dataset built around a maximal sampling of corbiculate bee genera would be most beneficial for further refining the potential affinities of this enigmatic fossil. In particularly, it would be very interesting to discover whether the jugal lobe of the hind wing was replaced by the distinctive jugal comb, a notable synapomorphy of Euglossini (e.g. [63]). Presently, there does not appear to be a jugal comb present but the hind wings are so poorly preserved that a definitive presence or absence cannot be stated. In the interim it is best to conservatively consider *E. biesmeijeri* as tribe *incertae sedis*.

Tribe: Bombini Latreille1802

Genus: *Bombus* Latreille 1802

**Bombus cerdanyensis** ***Dehon, De Meulemeester & Engel sp. nov.***

urn:lsid:zoobank.org:act:C61BA7C3-AD4E-4BA5-B277-4BA2F7AD798E

Holotype. Sex unknown. Conserved in the Palaeontology department collection, Muséum National d'Histoire Naturelle, Paris, France. The fossil consists in 2 parts: the compression and the imprint of the compression.

Etymology. The specific epithet is a reference to the lacustrine deposits of the la Cerdanya, Spain where the holotype was collected.

Type strata and locality. Late Miocene, lacustrine beds of Cerdanya, Spain.

Diagnosis. Forewing with alar papillae present on membrane beyond apical crossveins; membrane infuscated throughout, particularly in area beyond apical crossveins and along anterior borders of radial and marginal cells; pterostigma relatively small, trapezoidal, not greatly larger relative to prestigma and width not much shorter than length; marginal cell longer than distance from its apex to wing tip, tapering in width across its length, apex acutely rounded, not appendiculate, slightly offset from wing margin; three submarginal cells of relatively similar size (i.e., one not distinctly enlarged relative to the other two), anterior borders of second and third submarginal cells subequal; 1m-cu distinctly and strongly angulate anteriorly, meeting second submarginal cell near midpoint; 2m-cu weakly arched, meeting third submarginal cell in apical fifth; mesotibia five times longer than wide.

**Figure 4:**
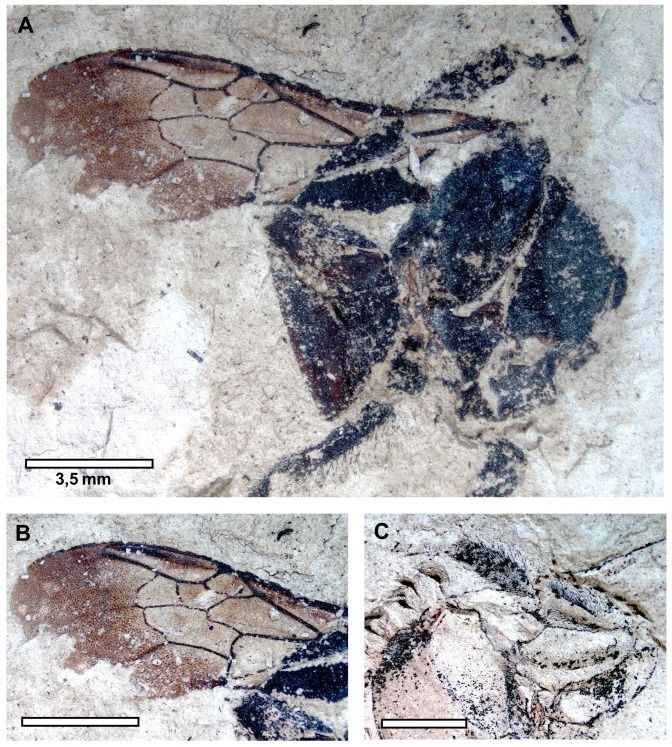
*Bombus cerdanyensis* sp. nov. A. General habitus. B. Detail of the left forewing. C. Detail of the middle leg on the imprint of the compression (photographs by T. De Meulemeester).

*Description. Sex unknown* (Fig. 4). A compressed individual in apparently dorsal oblique view with left forewing outstretched (right forewing not preserved); hind wing not preserved; head not preserved; mesosoma and metasoma largely incomplete and damaged; mid and hind legs preserved but somewhat jumbled and in some places partially overlapping forewing (e.g., obscuring origin of basal vein relative to 1cu-a but orientation of veins implies 1cu-a was apical basal vein origin). Right profemur 1.43 long, 0.88 wide as preserved (incomplete). Left mesofemur 3.60 mm long, 0.93 mm wide; mesotibia 3 mm long, 0.59 mm wide; mesobasitarsus 3.22 mm long, 0.91 mm wide; remaining tarsomeres and pretarsal claws well preserved, with apical most tarsomere longer than individual lengths of preceding tarsomeres; pretarsal claws not toothed as preserved. Right mesofemur 3.45 long, 0.47 wide; mesotibia 2 mm long, 0.38 wide as preserved (incomplete). Left forewing 13.25 mm long, 4.56 mm wide; three submarginal cells of relatively equal sizes; first submarginal cell 1.50 mm long (as measured from origin of Rs+M to juncture of r-rs and Rs), 0.67 mm high (as measured from Rs+M to pterostigma); second submarginal cell 1.52 mm long (as measured from juncture of Rs+M and M to juncture of Rs and 1rs-m), 0.78 mm high (as measured from midpoint on M between 1m-cu and 1rs-m to juncture of r-rs and Rs); third submarginal cell 1.32 mm long (as measured from juncture of 1rs-m and M to juncture of M and 2rs-m), 1.08 mm high (as measured from juncture of M and 2m-cu to juncture of 2rs-m and Rs); first medial cell 3.41 mm long (measured from juncture of M+Cu and Cu to juncture of 1m-cu and M), 1.16 mm high (measured from juncture of M and Rs+M to midpoint on Cu between M+Cu and 1m-cu); pterostigma 0.93 mm long; marginal cell 3.44 mm long, width tapering gradually across its length, apex rounded, offset from anterior wing margin, not appendiculate; 1m-cu distinctly and strongly angulate, meeting second submarginal cell near midpoint; 2m-cu weakly arched, meeting third submarginal cell in apical fifth. Metasoma 5.80 mm wide as preserved; first two segments visible, first segment 1.76 mm long, second segment 1.23 mm long (incomplete as preserved).

Comments. The LDA place the specimen among the corbiculate tribes. Therein, the venation is plesiomorphic relative to the Apini and Meliponini and in the same characters as highlighted for *Euglossopteryx* gen. nov. (*vide supra*). The relative sizes of the prestigma and pterostigma exclude a placement in the Electrobombini (although the presence or absence of a jugal lobe in the hind wing cannot be determined in the holotype). The forewing is apically papillate (as in Bombini), and the marginal cell is not appendiculate and 1m-cu is strongly angulate together suggesting the species does not belong to the Electrapini or Melikertini (although some melikertines have 1m-cu more angulate, such as *M. trigona*, 1m-cu is always much shorter and not as long as in Bombini or Euglossini; a long 1m-cu is more plesiomorphic among Corbiculata). Indeed, the forewings of the present fossil are distinctly *Bombus*-like: presence of papillae, general infuscation of the membrane, three submarginal cells of relatively similar size (albeit the latter character is assuredly plesiomorphic). As noted above a more refined analysis with the present fossil and a diversity of species of *Bombus* further reinforced the great similarity of the general wing shapes and supports inclusion within this genus, although attribution to a particular subgenus was not possible (refer to Results, above). Placement in *Bombus* is also in accordance with the geographic region and paleohabitat. Bumble bees are found throughout the Holarctic, Orient, and South America, but likely originated in the Palaearctic [65] and are principally associated with temperate and cold climates today [2], [66], [67]

Subfamily: Apinae Latreille 1802

Tribe: Anthophorini Dahlbom 1835

***Genus*** **Protohabropoda** ***Dehon & Engel gen. nov.***

urn:lsid:zoobank.org:act:809CFD9C-D3BD-4796-90E6-FECC65058AEC

Type species. *Protohabropoda pauli* De Meulemeester & Michez, new species.

Diagnosis. Female forewing with small pterostigma, pterostigma not tapering beyond r-rs, parallel-sided, length about equal to width; marginal cell broad, scarcely tapering apically, apex broadly rounded, slightly offset from anterior wing margin, not greatly extending beyond apical tangent of submarginal cells; three submarginal cells (i.e., 1rs-m present), first submarginal cell longer than individual lengths of second and third submarginal cells, but not longer than combined length of second and third submarginal cells; 1m-cu elongate, distinctly and strongly oblique, not angulate, relatively straight for most of its length, with weak arch in apical quarter (in this respect differing from *Habropoda*), meeting second submarginal cell near its apex (meeting cell only slightly basal to 1rs-m) (differing in this from *Anthophora* and *Amegilla*). Body of robust anthophoriform type, apparently densely setose (as in most modern Anthophorini); clypeus rounded.

Etymology. The new genus-group name is a combination of protos (Greek, meaning, “first”) and and the generic name *Habropoda*, the most similar extant genus to the fossil. The name is feminine.

**Protohabropoda pauli** ***De Meulemeester & Michez sp. nov.***

urn:lsid:zoobank.org:act:896FC513-0520-4EE0-A777-9CAEAFFBE5BC

Holotype. Female. Conserved in the Palaeontology department collection, Muséum National d'Histoire Naturelle, Paris, France. The fossil consists in 2 parts: the compression and the imprint of the compression.

Type strata and locality. Late Oligocene, lacustrine beds, Céreste, France.

Diagnosis. As for the genus (*vide supra*).

**Figure 5:**
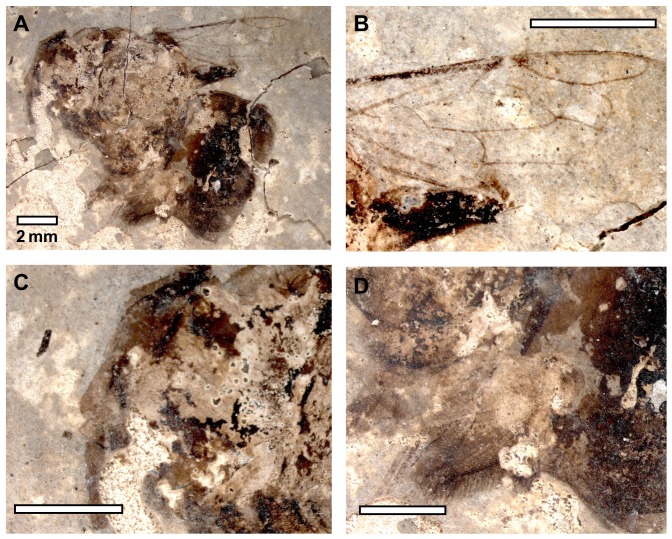
*Protohabropoda pauli* gen. nov. sp. nov. A. General habitus. B. Detail of wing. C. Detail of the head. D. Detail of the scopa (photographs by T. De Meulemeester).

Description. *Female* (Fig. 5). Dorsal-obliquely compressed individual, with apparently right forewing outstretched at oblique angle to body (hind wings not preserved); head turned with left lateral surface visible and depicting compound eye, gena, and face. Head with clypeus rounded; compound eye length 1.15 mm; left antenna preserved but incomplete, details of individual flagellomeres not discernible. Mesosoma 5.88 mm long; individual segments not discernible as preserved; dense pilosity present, some setae branched. Legs only partially preserved overlayed with mesosoma and slightly beneath; individual podites not measureable as preserved but distinctly with dense setation. Forewing 7.79 mm long, 2.53 mm wide; pterostigma small, 0.78 mm long, not tapering beyond r-rs, parallel-sided; marginal cell 1.98 mm long, 0.46 mm wide, scarcely tapering apically, apex broadly rounded, slightly offset from anterior wing margin, not greatly extending beyond apical tangent of submarginal cells; three submarginal cells (i.e., 1rs-m present); first submarginal cell 1.21 mm long (as measured from origin of Rs+M to juncture of r-rs and Rs), 0.45 mm high (as measured from Rs+M to pterostigma); second submarginal cell 0.75 mm long (as measured from juncture of Rs+M and M to juncture of Rs and 1rs-m), 0.69 mm high (as measured from midpoint on M between 1m-cu and 1rs-m to juncture of r-rs and Rs); third submarginal cell 0.71 mm long (as measured from juncture of 1rs-m and M to juncture of M and 2rs-m), 0.75 mm high (as measured from juncture of M and 2m-cu to juncture of 2rs-m and Rs); first medial cell 2.86 mm long (measured from juncture of M+Cu and Cu to juncture of 1m-cu and M), 0.71 mm high (measured from juncture of M and Rs+M to midpoint on Cu between M+Cu and 1m-cu); 1m-cu elongate, distinctly and strongly oblique, not angulate, relatively straight for most of its length, with weak arch in apical quarter, meeting second submarginal cell only slightly basal 1rs-m; 2m-cu weakly arched. Metasoma in lateral aspect, 4.5 mm long as preserved, 6.59 mm wide; two segments discernible; with dense pilosity over integument.

#### Male

Unknown.

Etymology. The specific epithet is a patronym honoring Mr. Paul Léon Victor Vigot, young scientist. In acknowledgment of his interest dedicated to bee systematics.

Comments. The shape of pterostigma (small, not tapering beyond r-rs, and parallel-sided) in *P. pauli* is similar to those of both the Anthophorini and Centridini. However, the ratio of the first (larger) and second (smaller) submarginal cells and the global shape of the forewing (Fig. 5) are more indicative of an anthophorine and the clypeus is rounded as in many Anthophorini. Seven extant genera are included in the Anthophorini: *Anthophora* (worldwide, 350 species), *Amegilla* (Old World, 250 species), *Deltoptila* (Central America, 10 species), *Elaphropoda* (Eastern Asia, 11 species), *Habrophorula* (Oriental, 3 species), *Habropoda* (Old and New World, 60 species), and *Pachymelus* (Southern Africa and Madagascar, 20 species) [68]. The new fossil specimen can easily be distinguished from *Anthophora* and *Amegilla* 1m-cu meeting the second submarginal cell near its apex, while the elongate and strongly oblique 1m-cu differs from that observed in *Habropoda*.

Family: Andrenidae Latreille, 1802

Subfamily: Andreninae Latreille, 1802

Genus: *Andrena* Fabricius, 1775

**Andrena antoinei** ***Michez & De Meulemeester sp. nov.***

urn:lsid:zoobank.org:act:EE404B56-22F5-4FB9-9075-B1FC5BBA2F05

Holotype. Male. Conserved in the Palaeontology department collection, Muséum National d'Histoire Naturelle, Paris, France.

Type strata and locality. Late Oligocene, lacustrine beds, Céreste, France.

Diagnosis. Male with yellow clypeus, remainder of head black; mesosoma black; legs and antenna brown; metasoma largely brown although black on apical segments (as preserved); wing membranes hyaline, veins brown; inner margins of compound eyes relatively straight, parallel (i.e., not converging anteriorly); forewing with basal vein confluent with 1cu-a, the latter strongly oblique; basal vein weakly arched in basal half; pterostigma more than three times longer than wide, tapering inside of marginal cell, border inside of marginal cell slightly convex; marginal cell long, tapering gently along length to acutely rounded apex, not appendiculate; first submarginal cell elongate, slightly longer than combined lengths of second and third submarginal cells; third submarginal cell greatly projecting apically in posterior half (resulting from strongly arcuate 2rs-m); 1m-cu meeting second submarginal cell beyond cell midpoint, relatively straight over much of length, then strongly angled to meet M; 2m-cu meeting third submarginal cell in apical third, straight; 1rs-m straight; 2rs-m strongly arcuate such that posterior border of third submarginal cell is nearly twice length of anterior border.

**Figure 6:**
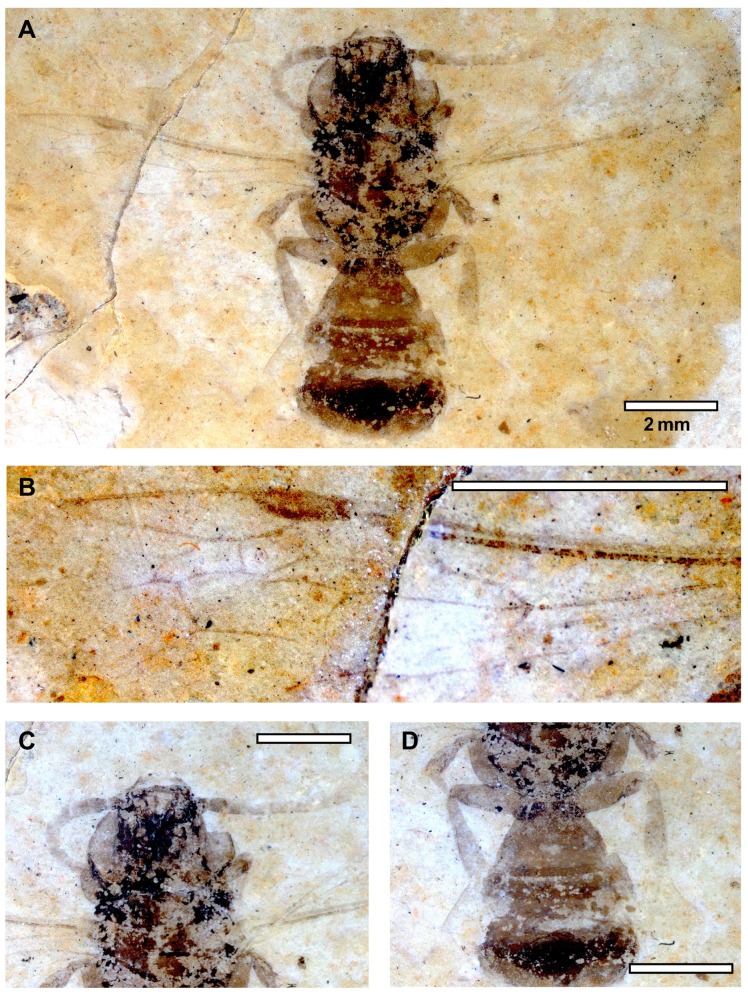
Male of *Andrena antoinei* sp. nov. A. General habitus. B. Detail of the wing. C. Detail of the head. D. Detail of the abdomen and the hind legs (photographs by T. De Meulemeester).

Description. *Male* (Fig. 6). Dorsal compressed individual, with head thrust forward and showing facial view, antennae extending laterally from body, left antenna curling back under head; dorsal view of mesosoma; forewings outstretched orthogonal to long axis of body; legs preserved along side of body; metasoma preserving basal few segments. Head 2.56 mm long, 2.30 mm wide; compound eyes 1.62 mm long, 0.53 mm wide; inner margins parallel; clypeus off white (likely yellow in life), 0.41 mm long, 0.57 mm wide; labrum 0.13 mm long; mandibles simple; right antenna incomplete, 0.26 mm wide; scape 0.4 mm long, pedicel 0.27 mm long, basal seven flagellomeres preserved, each 0.38 mm long; left antenna with only six articles preserved; terminal part of left antenna curled under the head. Mesosoma 2.90 mm long, intertegular distance 2.83 mm; mesoscutum 1.43 mm long; mesoscutellum 0.57 mm long; metanotum 0.25 mm long; propodeum 0.81 mm long (measured as preserved across dorsal and posterior surfaces). Profemur only partially exposed; left mesofemur 1.26 mm long, 0.41 mm wide; mesotibia 1.39 mm long, 0.37 mm wide; mesobasitarsus 0.50 mm long, 0.15 mm wide (incomplete); right mesofemur 1.20 mm long, 0.41 mm wide; mesotibia 0.96 mm long, 0.37 mm wide (incomplete); left metafemur 1.65 mm long, 0.55 mm wide; metatibia 1.85 mm long, 0.45 mm wide (incomplete); right metafemur 1.67 mm long, 0.54 mm wide; metatibia 1.81 mm long, 0.47 mm wide; metabasitarsus 1.34 mm long as preserved (incomplete). Forewing 5.94 mm long, 1.53 mm wide; pterostigma 0.72 mm long, 0.22 mm wide; marginal cell 1.62 mm long, tapering gently over length, apex acutely rounded; three submarginal cells, first submarginal cell 1.23 mm long (as measured from origin of Rs+M to juncture of r-rs and Rs), 0.28 mm high (as measured from Rs+M to pterostigma); second submarginal cell 0.50 mm long (as measured from juncture of Rs+M and M to juncture of Rs and 1rs-m), 0.26 mm high (as measured from midpoint on M between 1m-cu and 1rs-m to juncture of r-rs and Rs); third submarginal cell 0.66 mm long (as measured from juncture of 1rs-m and M to juncture of M and 2rs-m), 0.39 mm high (as measured from juncture of M and 2m-cu to juncture of 2rs-m and Rs); first medial cell 2.0 mm long (measured from juncture of M+Cu and Cu to juncture of 1m-cu and M), 0.29 mm high (measured from juncture of M and Rs+M to midpoint on Cu between M+Cu and 1m-cu); 1rs-m straight; 2rs-m strongly arcuate, thus projecting posterior half of third submarginal cell strongly apical with anterior border of second about one-half posterior border of cell; 1m-cu relatively straight for most of length; 2m-cu straight, 0.38 mm long. Metasoma 4.47 mm long as preserved, 3.10 mm wide; only basal few segments preserved, lighter in coloration than head and mesosoma, apparently with scattered minute setae.

#### Female

Unknown.

Etymology. The specific epithet is a patronym honoring Mr. Antoine Michez, in recognition of his dedication to melittology.

Comments. The morphometric analysis of forewing shape demonstrates that this species belongs to the Andrenidae (refer to Results, above). Unfortunately, diagnostic features of Andrenidae such as the two subantennal sulci below each antenna and the short to long-pointed glossa are not observable in the fossil. Nonetheless, the placement of the fossil in Andreninae is quite strong and the general habitus is certainly in accordance with such an assignment. The three submarginal cells and apically pointed marginal cell, with a slightly arched basal vein together exclude placement in any other subfamily of Andrenidae. The species does not exhibit any features which would permit its confident placement outside of the widespread and hyper diverse genus *Andrena* and so we have placed our species therein, pending the discovery of more complete material. The fossil further documents the presence of this lineage among the Late Oligocene fauna of France.

## Discussion

### Geometric morphometrics of wing shape to discriminate taxa

Michez *et al.* (2009) [31], De Meulemeester *et al.* (2012) [32], Wappler *et al.* (2012) [18], and Dewulf *et al.* (2014) [21] have shown that geometric morphometric analyses of forewing shape are a valuable tool for associating challenging bee fossils, particularly when other sources of character information are limited or lacking entirely. Naturally, it is most accurate and ideal to combine such geometric morphometric results with other anatomical traits for which cladistic polarity may be determined (e.g., leg morphology, mouthpart characters, etc.), but this is not always achievable with compression fossils such as the ones discussed herein. Thus, geometric morphometrics offers a way to open up a source of previously untapped data from such problematic specimens and thereby offer insights into bee evolution at different phases in their history. Indeed, these methods have proven useful in a variety of cases and lineages for discriminating taxa (e.g. [32], [69]–[71]). Here we show that bees can be discriminated based on their forewing shape at a variety of taxonomic levels, ranging from subfamily and above. Discriminant analyses employed in our study discriminated Anthophila from the families of apoid wasps, and even among the currently recognized families and subfamilies with reasonable robustness.

Morphological similarities at different taxonomical levels cannot be mistaken for phylogenetic relationships. They are merely similarities and this is highlighted by our own data. Exploration of our dataset representing significant parts of the bee forewing shape variation showed that phylogenetically close taxa are not always close in terms of overall similarity (e.g., Procrust distance is relatively high between Apidae and Megachilidae while these families are close phylogenetic relatives and among the families of Anthophila are actually sister) [1], [3], [72]. Despite these facts we observed a strong “taxonomic integrity” within forewing shape; i.e., all species were assigned to their corresponding tribe, all tribes were assigned to their corresponding subfamily, and all subfamilies were assigned to their corresponding family with high fidelity. We therefore argue that even though the morphological similarities do not represent phylogenetic relationships, there are patterns in forewing shape among members of individual clades. This statement is based on a supervised approach (i.e., LDA), so the assignment could be related to subtle common shape characteristics. Therefore we cannot exclude that only a part of the forewing shape may be responsible for this “taxonomic integrity”. Further fundamental studies on the phylogenetic signal of bee forewing shape, and morphometric integration and modularity in wing landmark configuration, remain to be undertaken and will certainly provide a considerable insight into apoid wing evolution. In fact, there are now well-established procedures for using morphometric data in cladistic analyses and this would be a fruitful line of investigation. In addition, such work might reveal those aspects of forewing shape tied to phylogeny and those more labile and perhaps tied to flight function and common to unrelated groups but whose flight mechanics are the same (e.g., correlates with body size, flight temperatures, etc.). This all highlights the broad potential for geometric morphometrics in evolutionary studies on bees.

### Corbiculate apine history

Corbiculate bees are among the most common of bees found as fossils, particularly in a variety of amber deposits [11], [73]. The clade is of Late Cretaceous age as evidenced by the occurrence of a crown-group meliponine in Maastrichtian-aged Raritan amber [61], [74], and regardless of the preferred phylogenetic place of Meliponini (i.e., sister to Apini or Bombini: Cardinal & Packer, 2007) [60] demonstrates that the cladogenetic events among the tribes extend back to at least the latest Cretaceous. A Cretaceous crown-group meliponine means that stem- or crown-group members of the other tribes must have been present in the latest Cretaceous and all are, therefore, quite old. The morphological isolation of the respective living tribes, combined with their antiquity, also emphasizes that various extinct groups must have existed that intermingle subsets of their traits and, in fact, such taxa have been found persisting until the Eocene (i.e., the last global greenhouse epoch). Already from the Eocene are known three extinct tribes and some of these were assuredly advanced eusocial like the Apini and Meliponini [1]. The discovery of the corbiculate *Euglossopteryx biesmeijeri* gen. nov. sp. nov. provides new insights into corbiculate diversity in the middle Eocene of North America. This species also suggests that the global cooling and drying that marked the Eocene-Oligocene transition and that was likely responsible for the loss of corbiculate diversity in Europe [1], [13], [15] was probably a global phenomenon and impacted similar bee groups in the New World.

Among the corbiculate lineages, the Bombini is the only tribe with a higher extant diversity in temperate and cold habitats. Like most bee groups, the fossil record of bumble bees is sparse [11], [18]. Twelve fossils have been documented from Oligocene through Miocene deposits as putative Bombini [18]; however, most of these fossils are poorly described and their placement within *Bombus* s.l. remains to be tested critically. Among those fossils, *B. cerdanyensis* and *B. randeckensis* have been the most thoroughly examined and in the case of the latter a confident subgeneric assignment has been demonstrated. These two specimens are of Late Miocene (∼10 Ma: the Spanish fossil) and Early Miocene (16–18 Ma: the German species), respectively, and both have broad implications for the dating of bumble bee evolution. For example, *Bombus randeckensis* was shown to belong to *Bombus* s.str., regardless of whether it should be placed as a stem group or within the crown group, and therefore provided direct evidence regarding the age of divergence between *Bombus* s.str. and *Alpinobombus* (i.e., minimally at 16–18 Ma) and in stark contrast to molecular estimates which grossly under-dated the clade [63]. *Bombus randeckensis* was, therefore, a direct observational example of how many age estimates may be significantly off (with crown-group *Bombus* s.str. being almost twice as old as available estimates) [18]. The subgeneric placement of *B. cerdanyensis* is less obvious than that of *B. randeckensis* but the two fossils are certainly not representative of the same subgeneric clade. Together these species demonstrate that *Bombus* had well diversified by the Miocene and that the group as a whole is much older than molecular-only methods might imply. Continued paleontological exploration will only further refine our understanding, based on direct evidence, of bombine evolution and timing.

### Anthophorine diversity and history

Multiple lines of evidence demonstrate the antiquity of the digger bees (Anthophorini) (e.g. [31], [75]). Indeed, the tribe certainly extends well into the Cretaceous and the most basal among extant genera (i.e., *Habrophorula* and *Elaphropoda*) are found in tropical Asia while derived genera such as *Anthophora* are more widely distributed in mesic and xeric habitats [67]. The distribution of those few fossil records attributable to Anthophorini seem to loosely confirm the ancestral adaptation of these bees to tropical habitats with subsequent invasion and diversification within mesic and xeric environments. *Paleohabropoda oudardi* was recorded from the Paleocene deposits of Menat, France (∼60 Myr) and which was at the time a distinctly tropical climate [31]. In contrast, the associated climate of the newly described *Protohabropoda pauli* gen. nov. sp. nov. was certainly mesic to xeric. It appears that the shift among anthophorines to non-tropical environments had already occurred during the Oligocene.

## Supporting Information

Table S1 **Landmarks coordinates of the Anthophorini data set (from Michez** ***et al.*** **2009).**
(XLSX)

Table S2 **Landmarks coordinates of the Bombini data set (from Wappler** ***et al.*** **2012).**
(XLSX)

Table S3 **Landmarks coordinates of the four new fossil specimens.**
(XLSX)

Table S4 **Landmarks coordinates of the 632 original specimens and the entomological collections from where they are loaned.**
(XLSX)

Table S5 **Mahalanobis distances (MD) between: (i) tribes centroids and the 632 specimens, (ii) the fossil** ***Euglossopteryx biesmeijeri*** **gen. nov. sp. n. and tribes centroids, (iii) the fossil** ***Bombus cerdanyensis*** **sp. nov. and tribes centroids, (iv) between the fossil** ***Protohabropoda pauli*** **gen. nov. sp. nov. and tribes centroids, (v) between the fossil** ***Andrena antoinei*** **sp. nov. and tribes centroids.**
(XLSX)

Table S6 **Specimen assignment in subfamilies using the cross-validation procedure in the LDA of forewing shape.** Original groups are along the rows, predicted groups are along the columns. The hit ratio (HR%) is given for each subfamily.
(XLSX)

Table S7 **Specimen assignment in tribes using the cross-validation procedure in the LDA of wing shape.** Original groups are along the rows, predicted groups are along the columns. The hit ratio (HR%) is given for each tribe.
(XLSX)

Table S8 **Specimens scores along the LDs (for the following taxonomic levels: suprafamily, family, subfamily and tribe).**
(XLSX)
